# RAB GTPases and SNAREs at the *trans*-Golgi network in plants

**DOI:** 10.1007/s10265-022-01392-x

**Published:** 2022-04-29

**Authors:** Emi Ito, Tomohiro Uemura

**Affiliations:** Graduate School of Humanities and Sciences, Ochanomizu University, Bunkyo-ku, Tokyo, 112-8610 Japan

**Keywords:** Membrane traffic, Post-Golgi transport pathways, RAB GTPases, SNAREs, *Trans*-Golgi network

## Abstract

Membrane traffic is a fundamental cellular system to exchange proteins and membrane lipids among single membrane-bound organelles or between an organelle and the plasma membrane in order to keep integrity of the endomembrane system. RAB GTPases and SNARE proteins, the key regulators of membrane traffic, are conserved broadly among eukaryotic species. However, genome-wide analyses showed that organization of RABs and SNAREs that regulate the post-Golgi transport pathways is greatly diversified in plants compared to other model eukaryotes. Furthermore, some organelles acquired unique properties in plant lineages. Like in other eukaryotic systems, the *trans*-Golgi network of plants coordinates secretion and vacuolar transport; however, uniquely in plants, it also acts as a platform for endocytic transport and recycling. In this review, we focus on RAB GTPases and SNAREs that function at the TGN, and summarize how these regulators perform to control different transport pathways at the plant TGN. We also highlight the current knowledge of RABs and SNAREs’ role in regulation of plant development and plant responses to environmental stimuli.

## Introduction

**Fig. 1:**
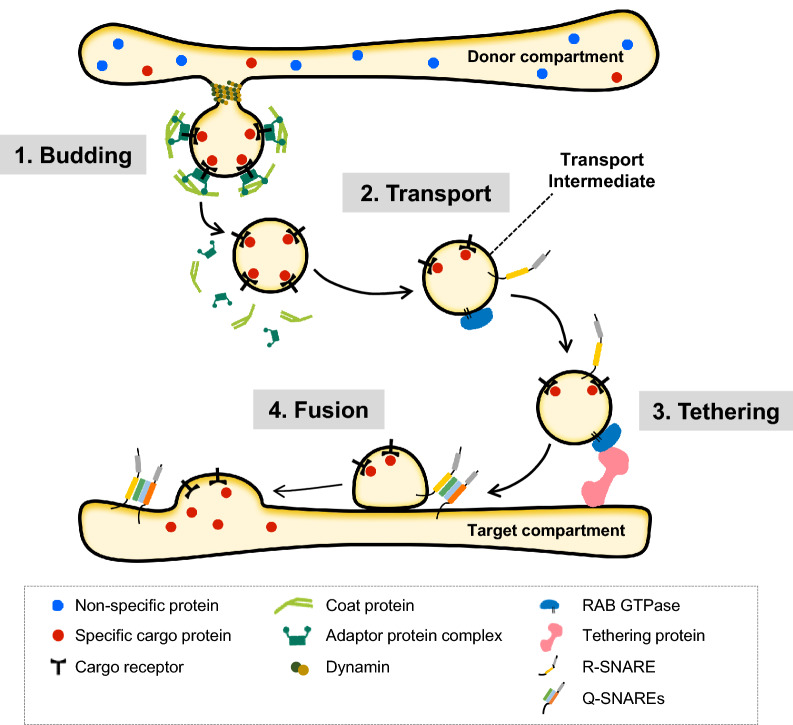
Process of membrane traffic. A single round of membrane traffic involves budding, transport, tethering and fusion steps that operate in sequence. Firstly, the soluble cargo in the lumen of the donor compartment is captured by the cargo receptor proteins. Then, the cytoplasmic tail region of the bound-cargo receptor interacts with adaptor proteins which in turn recruits coat proteins that deform the donor membrane so as to cage the cargo into a budding transport intermediate. Dynamin proteins are recruited to the narrow neck region of the bud and work like a lasso to pinch off the transport intermediate from the donor compartment. The coat protein and adaptor protein complex dissociate form the transport intermediate after budding. Next, the transport intermediate is delivered to the target compartment with the aid of motor proteins and cytoskeletons (not shown in this diagram). The transport intermediate is decorated with specific RAB GTPase and the R-SNARE protein. Once RAB finds the correct effector partner (tethering protein) that resides on the target compartment, RAB and effector together bridges the transport intermediate to the target compartment. Lastly, the R-SNARE on the transport intermediate forms *trans*-SNARE complex with the Q-SNAREs on the target membrane. The tight bundling of SNARE proteins leads to fusion of membranes of the transport intermediate and the target compartment, and subsequently, the cargo caged inside the transport intermediate is released to the lumen of the target compartment

Eukaryotic cells contain membrane-bound organelles that carry characteristic sets of proteins and membrane lipids, and correct placement of these specific components is critical to ensure functions of each organelle. The single membrane-bound organelles that constitute the endomembrane system [i.e., the endoplasmic reticulum (ER), Golgi apparatus, the *trans*-Golgi network (TGN), endosomes/multivesicular endosomes (MVEs), lysosomes/vacuoles and the plasma membrane (PM)] exchange proteins and membrane lipids among each other via process called membrane traffic. Membrane traffic exchanges molecules between the donor and target compartments by using membrane-bound intermediates (Fig. 1). This process was initially called “vesicular transport” since the transport intermediates are thought to be vesicular in shape; however, piling evidence showed that the transport intermediates can also be tubular or irregularly-shaped, therefore in this review, we refer to this process as “membrane traffic” rather than “vesicular transport”, and call the intermediate structures “transport intermediates”.

The process of membrane traffic is divided into four sequential steps: the first step is the “budding” step, in which the cargo at the donor organelle is sequestered into a specific domain on the donor membrane (also referred to as a “zone” of a membrane) and packaged into the transport intermediate. The second step is the “transport” step, in which the transport intermediate is delivered to the target compartment by motor proteins and cytoskeleton. In the third “tethering” step, the transport intermediate docks onto the target membrane, and subsequently the transport intermediate fuses with the target membrane thereby releasing the content to the target organelle during the last “fusion” step (Fig. 1). In addition, membrane lipids and proteins can travel to organelles with different identities as a result of a process called organelle maturation. A well-established example of such case is cisternal maturation of the Golgi apparatus. During cisternal maturation, the secretory cargo molecules travel with the Golgi cisternae without the need of being enveloped into the transport intermediates, and as the maturation process progresses, the cisternae itself changes its identity from *cis-* to *trans-*Golgi (Losev et al. 2006; Matsuura-Tokita et al. 2006). Furthermore, direct contacts between organelles also can transfer membrane lipids and proteins from one compartment to the other. One such example can be observed in yeasts where the secretory cargo is conveyed from the ER to the *cis-*Golgi via repeated approach (termed “hug-and-kiss” action) of *cis-*Golgi to the ER (Kurokawa et al. 2014).

**Fig. 2:**
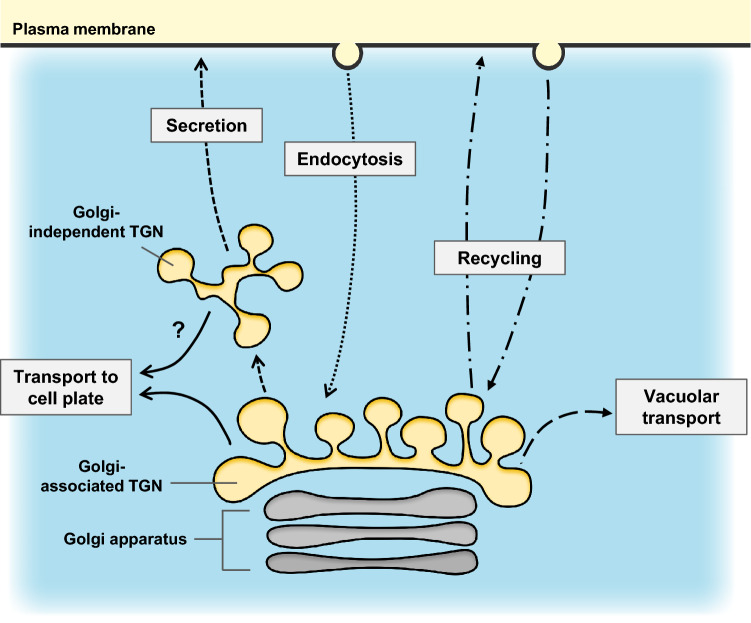
Plant TGN acts as a hub for secretion, endocytosis, recycling and vacuolar transport. Cargo proteins synthesized at the endoplasmic reticulum is transported to the Golgi apparatus, and then to the TGN. This transport process is called early secretion. At the TGN, the cargo is sorted and delivered to different destinations. Golgi-independent TGN, which is derived from the Golgi-associated TGN but located in distance from the Golgi-apparatus, is a specialized compartment for secretion (also referred to late secretion). Many of the trafficking components regulating secretion to the PM is utilized to deliver cargo to the cell plate. Recent study indicated that one Golgi-associated TGN bears “secretory-trafficking zone” at which the components of secretory machinery is accumulated and “vacuolar trafficking zone” at which the components regulating vacuolar transport is accumulated. Uniquely to plants, the TGN is the first compartment to which the endocytic cargo reaches, thus the plant TGN act as an early endosome that serves a platform for endocytosis and recycling. TGN; *trans*-Golgi network

The TGN, the tubulovesicular compartment adjacent to the *trans*-side of the Golgi apparatus, acts as an important hub that coordinates different trafficking routes to various target membranes in post-Golgi transport pathways. In eukaryotic cells, the TGN receives secretory and vacuolar cargo from the *trans*-cisternae of the Golgi apparatus, sorts and sends the cargo to the correct destination. Uniquely, in addition to this conserved role, the plant TGN is known to receive endocytic cargo from the PM and recycle selected cargo back to the PM (Fig. 2; Chow et al. 2008; Dettmer et al. 2006; Kang et al. 2011; Lam et al. 2007; Uemura et al. 2012a; Viotti et al. 2010). In animal cells, the endocytic cargo is received by a compartment separate from the TGN compartment called early endosomes (EE), whereas plants’ TGN is often designated as TGN/EE, as these two compartments share the same function. Interestingly, in comparison to the animal TGN which is tightly associated with the Golgi apparatus, the plant TGN can take a Golgi-independent state, in which the TGN is located further away from the Golgi apparatus, and behaves functionally independently from it (Kang et al. 2011; Uemura et al. 2014; Viotti et al. 2010). Recent findings suggest that such unique character of the plant TGN is strongly linked to the plant-specific organization of membrane traffic regulators. In this mini-review, we pick up two important regulators of membrane traffic, RAB GTPases and SNAREs, which are highly conserved among eukaryotic species but show unique features in plants, and highlight their functions at the TGN. We also summarize the involvement of these regulators in in plant development and environmental stress responses.

## RAB GTPases and their regulators at the TGN

RAB GTPase is a family belonging to the Ras GTPase superfamily, which members have ability to cycle between GTP-bound “active” and GDP-bound “inactive” states. When RAB is in the active state, it interacts with various effector proteins that include tethering complexes that dock transport intermediates to the correct target membrane (Grosshans et al. 2006; Zerial et al. 2001). Activation of RAB GTPases is regulated by guanine nucleotide exchange factors (GEFs) that catalyze exchange of GDP for GTP. Deactivation is regulated by GTPase-activating proteins (GAPs), which promote GTP hydrolysis activity of small GTPases, thereby accelerating hydrolytic reaction converting the GTP bound to RAB GTPase to GDP.

*RAB* genes are broadly conserved among eukaryotic species; Arabidopsis has 57 *RAB* genes that are classified into 8 groups (RAB1/RABD, RAB5/RABF, RAB6/RABH, RAB7/RABG, RAB8/RABE, RAB11/RABA, RAB2/RABB and RAB18/RABC), each of which is localized to distinctive organelles and marks different membranes of the endomembrane system (Table 1, Pereira-Leal et al. 2001; Rutherford et al. 2002; Ueda et al. 2002; Woollard et al. 2008). Interestingly, RAB11 group, which is known to regulate late secretory events in eukaryotic systems, is enormously diversified in plants. While humans and budding yeast have three (out of 66 RABs encoded in their genomes) and two (out of 11) RAB11s, respectively (Stenmark et al. 2001), Arabidopsis and rice have 26 (out of 57) and 15 (out of 39) RAB11/RABA members, respectively (Rutherford et al. 2002; Saito and Ueda 2009). In budding yeast, Ypt31/32 (the Rab11 orthologs) regulates secretory pathway by promoting vesicle formation at the TGN, and tethering of transport vesicles to the PM (Thomas et al. 2016). Mammalian Rab11s are shown to localize to the TGN, recycling endosomes (the endosomal compartments specialized for recycling proteins to the PM) and sorting endosomes (the early endosomal compartments in mammalian cells that receive endocytic cargo from the PM or the TGN, and sort and deliver the cargo to recycling endosomes, late endosomes/MVEs, the PM or the TGN), and regulate traffic of cargo from these compartments to the PM (Campa et al. 2017; Hsu et al. 2012; Naslavsky et al. 2018).

**Table 1:** Trafficking pathways regulated by plant RAB GTPases and their subcellular Localizations

	Pathways	Subcellular Localization	References
RAB1/RABD	ER-to-Golgi and ER-to-vacuole pathways	Golgi, TGN	Park et al. (1994), Kim et al. (1996), Hara-Nishimura et al. (1998), Batoko et al. (2000), Pinhero et al. (2009), Drakakai et al. (2012)
RAB2/RABB	ER-to-Golgi and ER-to-vacuole pathways	Golgi	Hara-Nishimura et al. (1998), Cheung et al. (2002)
RAB6/RABH	Golgi-to-ER pathway	Golgi, TGN	Bednarek et al. (1994), Johansen et al. (2009), Renna et al. (2018)
RAB11/RABA	Secretion, endocytosis, cell plate formation, (vacuolar transport?)	TGN, cell plate	Covered in this article
RAB8/RABE	Golgi-to-PM and ER-to-vacuole pathways	Golgi, PM	Hara-Nishimura et al. (1998), Zheng et al. (2005), Camacho et al. (2009); Speth et al. (2009), Mayers et al. (2017)
RAB5/RABF	MVEs-to-vacuole and MVEs-to-PM pathways	MVEs, PM	Ueda et al. (2001), Sohn et al. (2003), Kotzer et al. (2004), Ueda et al. (2004), Haas et al. (2007), Goh et al. (2007), Ebine et al. (2011), Sakurai et al. (2016), Ito et al. (2018)
RAB7/RABG	Vacuolar transport, ER-to-vacuole pathway	Vacuole	Hara-Nishimura et al. (1998), Saito et al. (2002), Cui et al. (2014), Ebine et al. (2014), Singh et al. (2014), Takemoto et al. (2018)
RAB18/RABC	(Endosomal pathway?)	?	

The uniqueness of the plant TGN functions may owe to this diversification of RAB11 members in plant lineages. Plant RAB11/RABA members are further classified into six subgroups: from RABA1 to A6, and most of them are shown to localized to the TGN or TGN-related structures. RABA1b, a member of the largest RABA1 subgroup, is demonstrated to localize to a distinctive region on the TGN (Asaoka et al. 2013a). RABA1b also colocalizes with VAMP721, R-SNARE (discussed later) that functions in the secretory pathway (Asaoka et al. 2013a), suggesting that RABA1b regulates secretory pathway from the TGN to the PM. Also, endocytic recycling of PIN1 from the TGN to the PM is impaired by *raba1b* mutation (Feraru et al. 2012), indicating that RABA1 is involved in recycling of cargo from the TGN to the PM. Interestingly, the *raba1a raba1b raba1c raba1d* quadruple mutant develops normally under a standard laboratory growth conditions; under salinity stress however, the quadruple mutant is stunted (Asaoka et al. 2013a, b). This suggests that RABA1-regulated secretion from the TGN is required for abiotic stress responses, such as salinity stress tolerance.

RABA2 and RABA3 also mark the distinctive domain on the TGN in non-dividing cells, and accumulate on the extending edges of the cell plates in dividing root cells (Chow et al. 2008). Lines of evidence suggest that plants use secretory and recycling machineries to deliver cell wall materials from the TGN to the newly forming cell plate during cytokinesis, and thus the traffic pathway from the TGN to the cell plate is termed “modified exocytosis” by Kanazawa et al. 2017). The Arabidopsis plants overexpressing the dominant negative mutant form of RABA2A are defective in cytokinesis (Chow et al. 2008), therefore, it is likely that RABA2 and RABA3 play major roles in modified exocytosis in dividing cells. RABA1b is also found on cell plates in dividing cells (Asaoka et al. 2013a); however, it was shown that RABA2a and RABA1e behaved differently when the cells were applied with endosidin 7 (ES7), an inhibitor of callose biosynthesis during cell plate formation (Davis et al. 2016; Park et al. 2014). Other study shows that *raba1*, *raba2* and *raba4* mutations affect different cell wall components (Lunn et al. 2013), suggesting that RABA2, RABA1 and RABA4 regulate different traffic pathways or transport different cargo during cell plate formation. Recent study indicated that RABA2 and RABA3, but not other RABA members, bind directly to SYP121 (PM-localizing Qa-SNARE) and VAMP721 (PM and TGN-localizing R-SNARE, discussed later) to regulate exocytosis (Pang et al. 2022). This also suggests RABA members are parts of different molecular machineries that coordinate secretion from the TGN.

A member of RABA4 group, RABA4b, is ubiquitously expressed in Arabidopsis. Meanwhile, the confocal laser scanning microscopy (CLSM) showed that in root hair cells, RABA4b is accumulated in the tips of the growing root hairs (Preuss et al. 2004). RABA4b interacts with phosphatidylinositol 4-kinase β1 (PI4Kβ1) and PI4Kβ2, when it is in active form (i.e., PI4Kβ1/2 are the downstream effectors of RABA4b). The mutations in *pi4kβ1* and *pi4kβ2* resulted in smaller plants with abnormal root hair shapes (Preuss et al. 2006), suggesting that RABA4 effectors regulate plant growth and root hair integrity downstream of RABA4. Likewise, RABA4d, the pollen-specific RABA4 member, is accumulated in the pollen tube tips, and *raba4d* mutation affected the shapes of pollen tubes and reduced their growth rates (Szumlanski et al. 2009). Tip growth involves massive secretion of proteins and polysaccharides exclusively to the tips of the growing cells (reviewed in Campanoni et al. 2007). Consistently, immunoelectron microscopy indicated that RABA4 and PI4Kβ1/β2 are predominantly localize to the secretory vesicle-forming region of the TGN (Kang et al. 2011; Preuss et al. 2006), suggesting that RABA4 regulates secretion from the TGN. These data together suggest that RABA4 members are involved in polarized secretion during tip growth. The members of RABA1 group are also shown to localize to the tips of the root hairs or the pollen tubes, for example, RABA1b and RABA1e are accumulated in the root hair tips, and RABA1f marks the tips of the pollen tubes (Asaoka et al. 2013a). Do RABA1 and RABA4 regulate the same trafficking events? It is shown that dominant negative mutant proteins of RABA1 and RABA4 exert different effects on the endocytosis of the PM-localized receptor, FLS2, upon ligand binding in tobacco expression system: while overexpression of dominant negative form of RABA1 impaired the correct localization of newly synthesized FLS2 to the PM, overexpression of dominant negative form of RABA4c accelerated endocytic transport of FLS2 to MVEs (Choi et al. 2013). This suggests that RABA1 and RABA4 regulate different trafficking steps at the TGN, although further studies are required.

A member of RABA5 group, RABA5c, is shown to accumulate on the growing edges of the cell plate during cytokinesis. In non-dividing cells, major population of RABA5c localizes to the large vesicles located near the PM of the geometric edges of the cells, and minor population is found to colocalize with the TGN marker (Kirchhelle et al. 2016). Overexpression of the dominant negative form of RABA5c resulted in disruption of lateral root shape owing to perturbation of the cell geometry by increasing the anisotropy of cortical microtubules and cellulose microfibrils (Kirchhelle et al. 2016, 2019). Taken together, it is proposed that RABA5 is responsible for regulating specific traffic pathway that sends materials from the TGN to the geometric edges of non-dividing cells to alter mechanical properties of cell edges.

In transient expression system using tobacco leaf epidermal cells, the overexpression of dominant negative form of RABA6a interfered with the traffic of endocytic cargo to the MVEs (Choi et al. 2013), thus RABA6 is suggested to regulate endocytic pathway. As far as we have surveyed, RABA6 is present sporadically in plant species. RABA6 is however present in Arabidopsis and *Amborella trichopoda*, while absent in *Oryza sativa*, *Selaginella moellendorffii*, *Physcomitrella patens*, and *Marchantia polymorpha*. This implies that RABA6 may have specialized roles in RABA6-possessing plants, though further study is needed to understand the exact roles of this subgroup.

RAB GTPases are activated by specific GEFs. Transport protein particles (TRAPP) family is a family of multi-subunit tethering complexes that activate specific RAB GTPase by acting as a GEF and tether transport intermediates to the correct target membrane (reviewed in Ravikumar et al. 2017; Vukasinovic et al. 2016). TRAPP family consists of four members (TRAPPI to IV) and each member is involved in different trafficking events in yeasts: TRAPPI is known to activate Ypt1 (ortholog of RAB1/RABD in plants) and mediate traffic from the ER to the Golgi apparatus, while TRAPPII is a GEF for Ypt31/32p (RAB11 ortholog in yeasts), and regulate post-Golgi traffic at the TGN (reviewed in Kim et al. 2016). TRAPPIII is also capable of activating Ypt1 more efficiently compared to TRAPPI (Thomas et al. 2018), suggesting that TRAPPIII plays a major role in regulating ER-to-Golgi trafficking. It is also reported that the specific component of TRAPPIII and TRAPPIV, namely Trs85 and Trs33, respectively, are involved in regulating Ypt1-mediated autophagy in yeasts (Lipatova et al. 2016; Lynch-Day et al. 2010).

Proteomic analysis indicated that the components of TRAPPI, II and III family members are present in purified TGN fraction (Drakakaki et al. 2012). The mutation in TRAPPI component caused mild defect in cytokinesis, whereas the mutation in TRAPPII component caused severer phenotypes during cytokinesis leading to defects in embryogenesis or seedling lethality (Qi et al. 2011; Thellmann et al. 2010). Also, the mutations in TRAPPII components caused abnormal accumulation of secretory markers inside the root cells (Qi et al. 2011). TRAPPII colocalizes with RABA1c, and mutation in TRAPPII component cause partial diffusion of RABA1c to cytosol (Qi et al. 2011), therefore, it is suggested that TRAPII functions upstream of RABA1c to regulate secretion from the TGN. A component of TRAPPII was also identified by a forward genetic screen aimed at isolating mutants that are defective in leaf venation (Naramoto et al. 2014). *VAN4*, *VASCULAR NETWORK DEFECTIVE 4*, coded TRS120, the specific component of TRAPPII complex, and *van4* mutant shows defects recycling of PIN proteins (Naramoto et al. 2014). VAN4/TRS120 colocalized with the TGN marker and RABA1c (Naramoto et al. 2014), further supporting that TRAPPII functions together with RABA1 to regulate recycling and secretory processes at the TGN. Proteomic analysis indicated that TRS120 co-precipitated with dominant negative form of RABA2a, and mutation in *trs120* interfered with the accumulation of RABA2a on the cell plate (Kalde et al. 2019), implying that TRAPPII also acts as a GEF for RABA2 members.

Interestingly, in the proteomic analysis of purified TGN followed by live cell imaging showed that RABD/RAB1 partially colocalizes with the TGN markers in plant cells (Drakakaki et al. 2012; Pinheiro et al. 2009). Mammalian and yeast homologs of RABD (Rab1 and Ypt1, respectively) participate in ER-to-Golgi trafficking, and plant RABD members, too, are reported to take part in this early secretory event (Batoko et al. 2000). Arabidopsis YIP1 was also identified in the proteomic analysis of purified TGN (Drakakaki et al. 2012). Yeast Yip1p (YPT/RAB GTPase Interacting Protein 1) is known to interact with inactive form of Ypt1/RAB1 and Ypt31/RAB11, and recruit Ypt1p to the Golgi membrane (Yang et al. 1998). In yeast, Ypt1, Ypt6 (RAB6/RABH ortholog), Ypt31/32p, Sec4p (RAB8/RABE ortholog) as well as their GEFs and GAPs act in cascade to ensure directional transport of secretory cargo from the ER-Golgi apparatus to the TGN (called “early secretion”), and then from the TGN to the PM (called “late secretion”) (Ortiz et al. 2002; Rivera-Molina et al. 2009; Suda et al. 2013; Wang et al. 2002). Several studies show that plant RAB6/RABH localizes mainly to the Golgi apparatus but subpopulation of RABH is found on the TGN (Johansen et al. 2009; Renna et al. 2018). This suggests that RABH may function during late secretion. Taken together, presence of plant RABD, YIP1 and RABH at the TGN implicates that RAB GTPase cascade regulates early and late secretion in plant system.

RABF/RAB5 is the key regulator of the endosomal trafficking. RAB5 is conserved broadly in eukaryotic organisms; however, land plants and some green algae species possess plant-unique RAB5, called ARA6/RABF1, in addition to the conventional RAB5s (RABF2A/RHA1 and RABF2B/ARA7 in Arabidopsis) (Ebine et al. 2011; Hoepflinger et al. 2013; Ueda et al. 2001, 2004). Both canonical RAB5s and ARA6 localize predominantly to the limiting membrane of MVEs (Haas et al. 2007; Scheuring et al. 2011); however, quantitative analysis indicated that canonical RAB5s and ARA6 are located in close proximity to the TGN marked by RABA1b, clathrin or SYNTAXIN OF PLANT 43 (SYP43; discussed later) (Asaoka et al. 2013a; Ito et al. 2012, 2016). Electron microscopy indicated that MVEs are often found in vicinity of the TGN (Kang et al. 2011), and immunoelectron microscopy showed that anti-RABF2B antibody labels both MVEs and the TGN (Stierhof et al. 2010). Live cell imaging also showed that ARA7 marks subdomain of the TGN (Singh et al. 2014), and ARA6 overlaps with the TGN marker when expressed under a strong promoter in tobacco leaf cells (Bottanelli et al. 2012). In addition, plants’ RAB5 activator, VPS9A (VACUOLAR SORTING PROTEIN 9A; Goh et al. 2007), is suggested to localize to the MVEs, as well as to the TGN (Sunada et al. 2016).

What is the implication of localization of RAB5s on the TGN? Ultrastructural studies and live-cell imaging showed that the MVE number was reduced when the TGN function was impaired by concanamycin A (V-ATPase inhibitor) (Scheuring et al. 2011), suggesting that MVE biogenesis is linked to the TGN function. In addition, an ultrastructural study also detected multivesiculated TGN-like structures, and live cell imaging showed that a hybrid compartment bearing both TGN and MVE markers are formed when dominant negative mutant protein of one of the ESCRT-III components is overexpressed in protoplasts (Scheuring et al. 2011). Another study also shows that PM-localizing FLS2 is transiently sequestered to the TGN-MVE hybrid compartment when endocytosis and vacuolar transport of FLS2 is triggered by ligand-binding in tobacco cells (Choi et al. 2013), implying that endocytic cargo passes the TGN-MVE hybrid structures en route to the vacuole. Based on these data, it is commonly thought that in plant system, MVEs are formed by multivesiculation and organelle maturation of the TGN. It is not clear if plant RAB5s have active roles in endosomal maturation of the TGN; nevertheless, it is likely that RAB5 marks the early phases of endosomal maturation.

## SNARE proteins at the TGN

SNARE (soluble *N-*ethylmaleimide-sensitive factor attachment protein receptors) proteins regulate fusion between a transport intermediate and its target membrane. SNAREs are grouped into Q- and R-SNAREs, which localize to the target membrane and the vesicular membrane (i.e., the membrane of the transport intermediate), respectively (Fasshauer et al. 1998). Q-SNAREs are further classified into Qa-, Qb- and Qc-SNAREs based on their amino acid sequences (Fasshauer et al. 1998). After transport intermediates are docked onto the target organelle, R-SNARE on the transport intermediate interacts with the cognate Qabc-SNAREs on the target membrane to form tight helical bundle called *trans*-SNARE complex. Subsequently, the target and vesicular membranes are brought closely, removing the water molecules between the juxtaposing membrane leaflets, and then the fusion occurs between the membranes. In Arabidopsis, at least 66 Q- and R-SNARE proteins are encoded in genome (Saito and Ueda 2009). The systematic localization analysis using Arabidopsis cultured cells has been done to map the subcellular localization of plant SNARE proteins (Uemura et al. 2004). The result firstly indicated that most of SNAREs are ubiquitously expressed in plants, and secondly that SNAREs mark distinctive organelles in plant cells.

Immuno-electron microscopy and systematic localization analysis indicated that plant TGN bears three Qa-SNAREs (SYNTAXIN OF PLANT (SYP) 41/42/43), three Qb-SNAREs (VPS TEN INTERACTING (VTI) 11/12/13) and one Qc-SNARE (SYP61) (Bassham et al. 2000; Kang et al. 2011; Sanderfoot et al. 2001; Uemura et al. 2004). Mutations in *SYP4* group impact secretory and vacuolar transport, as well as the morphology of Golgi apparatus and the TGN (Uemura et al. 2012a). Interestingly, uptake of lipophilic dye, FM4-46, is unaffected in *syp4* mutations (Uemura et al. 2012a), suggesting that constitutive endocytosis of membrane materials is not impaired by *syp4*. The *syp42 syp43* double mutant shows pleiotropic phenotypes. For example, the *syp42 syp43* double mutant is smaller in size, and shows aberrant response to gravity due to the loss of polar localization pattern of PIN2 proteins in root cells (Uemura et al. 2012a). The *syp42 syp43* double mutant is sensitive to abiotic stresses, such as salinity and osmotic stresses (Uemura et al. 2012b). In addition, the *syp42 syp43* double mutant is susceptible to non-host species of powdery mildew pathogen, and shows severe chlorosis (i.e. hyper-sensitive response) when infected with host species of powdery mildew fungus (Uemura et al. 2012a). The proteomic analyses using apoplastic cell fraction indicated that *syp42 syp43* double mutant is defective in secreting cell-wall modification enzymes to the apoplast when infected with the pathogen (Uemura et al. 2019). The *syp42 syp43* double mutant is defective in targeted secretion of VAMP721 (discussed later) to the plant-pathogen contact site, the process required for the plant to restrict pathogen entry (Uemura et al. 2019). These data together indicate that secretion and vacuolar transport of specific cargo regulated by SYP4 at the TGN is essential for normal development of plants, as well as, plant responses to environment and surrounding pathogens/microbes.

The live cell imaging showed that VTI11 and VTI13 localize to the TGN, MVEs and the vacuolar membrane, whereas VTI12 localizes on the TGN, MVEs and the PM in Arabidopsis protoplasts and root cells (Niihama et al. 2005; Uemura et al. 2004). VTI11 is capable of forming *trans*-SNARE complex with tonoplast-localized SNAREs, namely SYP22 (Qa) and SYP51 (Qc), as well as VAMP727 (R) that is involved in vacuolar trafficking (discussed below) (Ebine et al. 2008; Sanderfoot et al. 2001; Yano et al. 2003), suggesting that VTI11 regulates vacuolar transport. A forward genetic screen identified *vti11* mutant as *shoot gravitropism* 4 (*sgr4*)/*zigzag* (*zig*), which shows defects in shoot gravitropism (Kato et al. 2002; Morita et al. 2002). In endodermal cells of *vti1/zig*-*1* mutant, the amyloplasts (the statocysts) are abnormally accumulated on the upper side of the cell against gravity (Morita et al. 2002; Yano et al. 2003), suggesting that VTI11 is required for normal sedimentation of amyloplasts in the direction of gravity. The *vti11/zig-1* mutation also caused fragmentation of the vacuoles (Morita et al. 2002; Yano et al. 2003). Interestingly, single amino acid substitution mutation in *VTI12* gene suppressed the phenotype of *vti11/zig-1* mutant (Niihama et al. 2005). This mutation caused VTI12 to localize to the vacuole, and enabled VTI12 to form complex with SYP22 (Niihama et al. 2005). While a proteomic analysis showed that both VTI11 and VTI12 co-precipitate with GFP-tagged SYP43 (Fujiwara et al. 2014), other study indicated that VTI12 protein, but not VTI11, interacts with TGN-localizing SNAREs (i.e., SYP61 and SYP4) and VPS45 that regulate SNARE complex formation at the TGN (discussed later) (Bassham et al. 2000; Sanderfoot et al. 2001). In addition, a genetic data suggested that VTI11 and VTI12 regulate transport to lytic vacuole and storage vacuole, respectively (Sanmartin et al. 2007), and a detached leaf assay shows that *vti12* mutant exhibited severer chlorosis compared to wild-type and *vti11/zig-1* (Surpin et al. 2003). These data suggest that although the molecular functions of VTI11 and VTI12 are interchangeable, VTI11 and VTI12 may take part in distinctive traffic events. The traffic pathway that VTI13 regulates is not clear. VTI13 is localized to tonoplasts and punctate structures labeled by FM4-64 in Arabidopsis root hair cells, and shown to take part in root hair growth (Larson et al. 2014). Interestingly, ultrastructural analysis indicated that in *vti13* mutant cells, SYP4 is mislocalized to the ER (Larson et al. 2014), implying that VTI13 is required for the correct localization of SYP4 to the TGN.

*SYP61* gene was identified as *OSMOTIC STRESS-SENSITIVE MUTANT 1* (*OSM1*) by a genetic screen aimed at finding genes responsible for salinity stress responses (Zhu et al. 2002). The *syp61/osm1* mutant wilts easily compared to wild type when grown on soil with limited moisture, and is sensitive to salt and osmotic stresses (Zhu et al. 2002). SYP61 is shown to interact with a member of aquaporins called PLASMA MEMBRANE INTRINSIC PROTEIN 2;7 (PIP2;7) (Hachez et al. 2014). PIP2;7 localizes to the PM in wild-type root cells; however, in *syp61/osm1* mutant, PIP2;7 accumulated to ER-derived globular/lenticular structures (Hachez et al. 2014), suggesting that SYP61 is required to transport PIP2;7 to the PM via secretory pathway. Recent study indicates that SYP61 is ubiquitinated by a ubiquitin ligase ATL31, which is involved in carbon/nitrogen nutrient stress responses (Hasegawa et al. 2022; Sato et al. 2009). The *syp61* knock down mutant and *syp61/osm1* mutant are hypersensitive to C/N-nutrient imbalances (Hasegawa et al. 2022), suggesting that ubiquitination status of SYP61 is important for the response to nutrient availability.

Sec1/Munc-18 (SM) family proteins activate Qa-SNAREs, and promote SNARE complex assembly (Shen et al. 2007). Immunoelectron microscopy indicated that the SM protein, VPS45, localizes to the TGN, and coprecipitate with TGN-localized SNAREs but not with the MVE-localized SNAREs (Bassham et al. 2000). *VPS45* was also identified as *BEN2* (*BFA-visualized endocytic trafficking defective 2*) during a forward genetic screen aimed at isolating mutants that are defective in accumulation of PIN1 inside BFA bodies after BFA treatment (Tanaka et al. 2013). The *vps45/ben2* mutant produced smaller BFA bodies labeled by PIN1-GFP, and caused delay in endocytosis monitored by the tracer molecule, FM4-64 (Tanaka et al. 2013). This suggests that the activation of TGN-localizing SNAREs by VPS45 influences endocytosis and recycling of cargo, such as PIN1.

In yeasts and mammals, Ykt6, the R-SNARE, regulates versatile trafficking events, such as retrograde trafficking from the *cis-*Golgi to the ER, secretion, endosomal trafficking and vacuolar transport (reviewed in Kriegenburg et al. 2019). Also, YKT6 is shown to function in the process of fusion between autophagosome and lytic compartment in yeast, fly and mammals (Kriegenburg et al. 2019). Plant orthologs of Ykt6, YKT61 and YKT62 in Arabidopsis, interact with SYP41, and facilitates fusion between liposomes containing either SYP41 or SYP61 (Chen et al. 2005). This suggested that plant YKT6 is a putative R-SNARE regulating the trafficking at the TGN; however, the subcellular localization analysis indicated that GFP-tagged YKT6 is found in the cytosol in Arabidopsis protoplasts (Uemura et al., 2004), and thus the role of YKT6 at the plant TGN is still unclear. Recently, *ykt6* mutants produced by genome editing were shown to be defective in male and female gametogenesis and embryonic development (Ma et al. 2021). Ma et al (2021) had demonstrated that GFP-tagged YKT61 expressed under the regulation of the *UBIQUITIN10* promoter was associated to membranes, and distributed to cytosol and the punctate structures in Arabidopsis root cells. They also demonstrated that GFP-YKT61 interacts with SEC22 (ER-localizing R-SNARE), SYP22 (tonoplast-localizing Qa-SNARE), SYP41 (TGN-localizing Qa-SNARE, described above), VAMP721/722 (PM and TGN-localizing R-SNARE, discussed below) (Ma et al. 2021), altogether suggesting that plant YKT6 plays roles in multiple trafficking events, which may include the trafficking from and to the TGN.

The systematic analysis demonstrated that the members of VESICLE-ASSOCIATED MEMBRANE PROTEIN 72 (VAMP72), the R-SNAREs, are localized to the TGN and the PM in Arabidopsis protoplasts (Uemura et al. 2004). VAMP72 group consists of seven members (VAMP721 to VAMP727) among which the functions of VAMP721, VAMP722 and VAMP727 are extensively studied. In addition to the TGN and the PM, VAMP721 and VAMP722 localize to the cell plates in dividing cells (El Kasmi et al. 2013; Shimizu et al. 2021; Uemura et al. 2012a; Zhang et al. 2011), and are shown to regulate secretion from the TGN (Kwon et al. 2008; Shimizu et al. 2021). The *vamp721 vamp722* double mutation results in dwarf seedlings that show defects in cell plate formation and auxin-related responses (Zhang et al. 2011; Zhang et al. 2021). In *vamp721 vamp722* double mutant cells some of the PM markers and PIN proteins are mislocalized to the intracellular structures (Zhang et al. 2011; Zhang et al. 2021). Also, the *vamp721 vamp722* double mutation reduces the TGN number, affects the TGN size, and causes aggregation of the TGN (Zhang et al. 2021), suggesting that VAMP721 and VAMP722 are required to keep the integrity of the TGN. In addition, VAMP721 and VAMP722 are demonstrated to take part in plant immunity response to powdery mildews (Kwon et al. 2008), and to bacteria (Kim et al. 2021; Kwon et al. 2020; Yun et al. 2013). It is demonstrated that VAMP722 is accumulated at the powdery-mildew entry sites, and forms complex with PM-localizing Q-SNAREs, SYP121/PEN1 (PENETRATION 1) and SNAP33 (SYNAPTOSOMAL-ASSOCIATED PROTEIN OF 33 kDa) (Kwon et al. 2008) to secrete powdery-mildew resistance protein RPW8.2 (Kim et al. 2014). The VAMP721/722-depleted plants that were treated with bacterial elicitor accumulate enzyme of the lignin biosynthetic pathway, caffeoyl-CoA *O*-methyltransferase 1 (CCOAOMT1) inside the cells (Kwon et al. 2020), suggesting that VAMP721 and VAMP722 is responsible for secreting enzymes that are involved in cell wall reinforcement when a plant is infected with pathogenic bacteria. Recent study showed that VAMP721 interacts with PICALM1a/ECA1 (PHOSPHATIDYLINOSITOL-BINDING CLATHRIN ASSEMBLY 1A/EPSIN-LIKE CLATHRIN ADAPTOR 1) and PICALM1b, the ANTH-domain containing clathrin adaptor protein (Fujimoto et al. 2020). In *picalm1a/b* double mutant, VAMP721 is mislocalized to the PM, and the *picalm1a/b* double mutant is defective in secretion of mucilage after the imbibition (Fujimoto et al. 2020). Therefore, it is proposed that VAMP721 is recycled back to the TGN in PICALM1a/1b-dependent manner, and the recycling of VAMP721 is important for correct secretion of cargo, such as mucilage.

Unlike other VAMP72 members, the systematic analysis indicated that VAMP727 localizes to MVEs in Arabidopsis protoplast cells (Uemura et al. 2004). In planta, VAMP727 colocalizes mainly with MVE markers (Ebine et al. 2008); however, subpopulation of VAMP727 localizes to the TGN (Shimizu et al. 2021; Uemura et al. 2019). VAMP727 localized to the PM when endocytosis was blocked by PI3K-PI4K inhibitor, wortmannin, and VAMP727 further accumulates at the PM as a result of overexpression of constitutive active form of ARA6 (Ebine et al. 2011), suggesting that subpopulation of VAM727 cycles between the PM and ARA6-localizing MVEs. Biochemical analysis indicated that VAMP727 forms *trans*-SNARE complex with tonoplast-localized Q-SNAREs (Ebine et al. 2008), as well as the PM-localized Qa-SNAREs (Ebine et al. 2011), suggesting that VAMP727 is involved in versatile traffic pathways linking TGN-vacuole via MVEs, and MVE-PM. Meanwhile, VAMP727 is suggested to function mainly in vacuolar transport, since the mutation in *VAMP727* aggravates the phenotype of the *syp22-1* (mutant of tonoplast-localizing Qa-SNARE), and overexpression of VAMP727 recures the phenotype of the *syp22-1* (Ebine et al. 2008). Consistently, VAMP727 shares the same zone with components of vacuolar trafficking machineries, but not with components of the secretory traffic machineries on the TGN (Shimizu et al. 2021). Adaptor protein (AP) complex are known to bind to specific sorting signals in the cytosolic tail of the cargo and promote packaging of cargo to transport intermediates at the donor compartment. In other words, specific AP complex marks a distinctive zone of the organelle where the cargo for the specific traffic pathway is accumulated. AP-1 is shown to take part in secretory pathway and trafficking pathway to the cell plate, whereas AP-4 is known to interact with the vacuolar sorting receptors and takes part in vacuolar transport (Fuji et al. 2016; Singh et al. 2018). Shimizu et al. have elegantly shown using super-resolution confocal live imaging microscopy (SCLIM) they had developed that a single TGN bears “secretory-trafficking zone” which is comprised of AP-1, clathrin and VAMP721, and “vacuolar trafficking zone” comprised of AP-4 and VAMP727 (Shimizu et al. 2021). In addition, 4 dimensional live-cell imaging demonstrated that a membrane fraction containing VAMP721, AP-1 and clathrin separates from the Golgi-associated TGN (GA-TGN) to produce Golgi-independent population of the TGN (termed GI-TGN; Shimizu et al. 2021; Uemura et al. 2014, 2019), suggesting that GI-TGN is the specialized population of the TGN that is responsible for secretion.

## Future perspectives

**Fig. 3:**
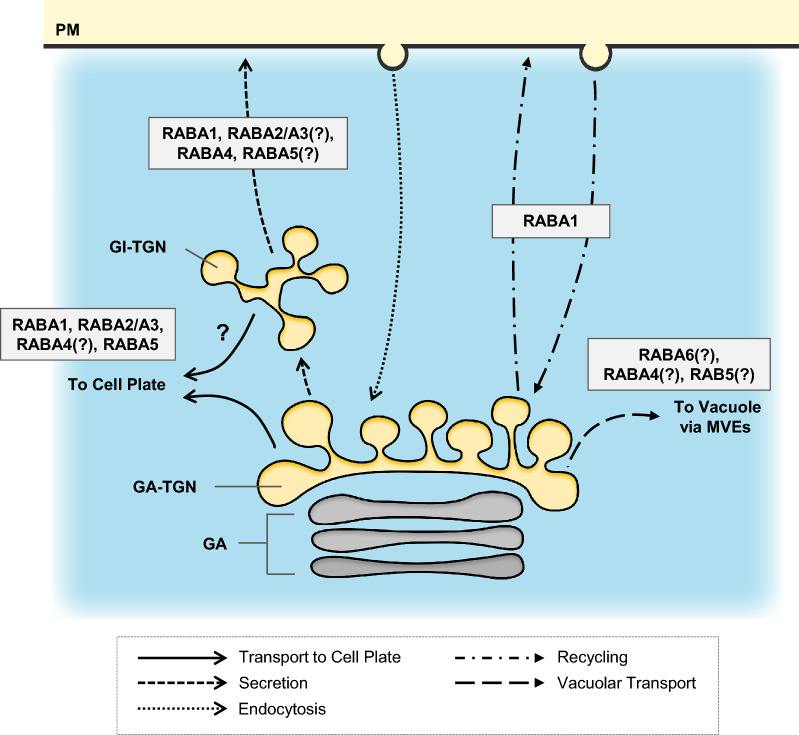
Roles of TGN-localizing RAB GTPases. Members of RABA/RAB11 group regulate different transport pathways form/to the TGN. RABA1 and RABA4 are suggested to regulate secretion, while RABA1 and RABA2/A3 are involved in transport to the cell plate. RABA1 is also suggested to regulate recycling of the PM-localizing membrane proteins. The precise member that regulates vacuolar transport from the TGN is not clear; however, lines of evidence suggests that RABF/RAB5 marks early phases of endosomal maturation. GI-TGN; Golgi-independent TGN, GA-TGN; Golgi-associated TGN, GA; Golgi apparatus, MVEs; multivesicular endosomes

**Fig. 4:**
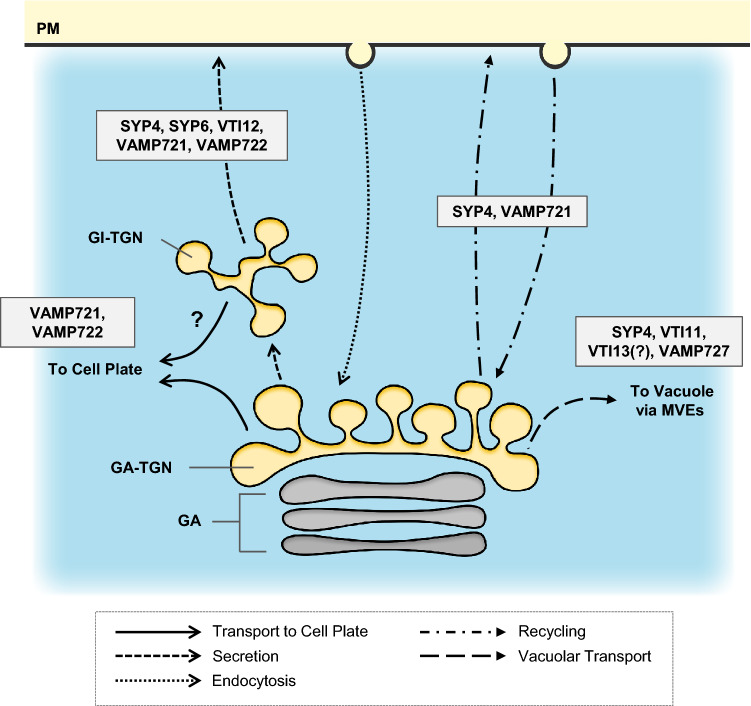
Roles of TGN-localizing SNAREs. SYP4, the Qa-SNARE, regulates secretion, recycling and vacuolar transport. Interestingly, SYP4 is not involved in constitutive endocytosis. Lines of evidence suggest that VTI12 takes part in the traffic pathway form the TGN to the PM, while VTI11 takes part in vacuolar transport form the TGN. SYP6, the Qc-SNARE, is shown to regulate secretion. VAMP721 and VAMP722 are involved in secretion, recycling and transport to the cell plate. VAMP721 is recycled back to the TGN by the action of PICALM1a/1b. VAMP727 mainly regulates vacuolar transport. VAMP721 and VAMP722 are the components characterizing the “secretory-trafficking zone”, while VAMP727 is the component characterizing the “vacuolar trafficking zone” of the TGN. GI-TGN; Golgi-independent TGN, GA-TGN; Golgi-associated TGN, GA; Golgi apparatus, MVEs; multivesicular endosomes

Plant TGN serves an important platform to coordinate secretion, recycling, vacuolar transport and endocytosis, and the trafficking of cargo via TGN is essential for plant development and plant response to various environmental stresses and stimuli. Figures 3 and 4 summarize the current understandings of roles of RAB GTPases and SNAREs in regulation of distinctive transport pathways from/to the TGN. Although the knowledge of the functions of RABs and SNAREs at the TGN is expanding, essential questions are still unanswered. For example, do different RABA members regulate distinctive trafficking routes, or do they share common effectors, trafficking machineries or functional “zones” to deliver different cargo to the designated destinations? What are the molecules responsible for demarking different trafficking zones on the same TGN? What exactly is the cargo delivered from the TGN during plant development, cell plate formation, abiotic/biotic stress responses? To answer to these questions, we believe that in addition to the classical cell biological approaches, application of latest techniques such as super resolution microscopy and artificial intelligence technology based on machine learning will allow us to detect and classify the fine dynamics of membrane trafficking regulators, and will be a key to deepen our understanding of the complex but unique functions of TGN in plant system.
