# Correction to: RAB GTPases and SNAREs at the *trans*-Golgi network in plants

**DOI:** 10.1007/s10265-022-01403-x

**Published:** 2022-06-11

**Authors:** Emi Ito, Tomohiro Uemura

**Affiliations:** Graduate School of Humanities and Sciences, Ochanomizu University, Bunkyo-ku, Tokyo, 112-8610 Japan

## Correction to: Journal of Plant Research (2022) 135:389–403 10.1007/s10265-022-01392-x

The article “RAB GTPases and SNAREs at the *trans*-Golgi network in plants”, written by Emi Ito, Tomohiro Uemura, was originally published Online First without Open Access. After publication in volume 135, issue 3, pages 389–403 the author decided to opt for Open Choice and to make the article an Open Access publication. Therefore, the copyright of the article has been changed to © The Author(s) 2022 and the article is forthwith distributed under a Creative Commons Attribution 4.0 International License, which permits use, sharing, adaptation, distribution and reproduction in any medium or format, as long as you give appropriate credit to the original author(s) and the source, provide a link to the Creative Commons licence, and indicate if changes were made. The images or other third party material in this article are included in the article’s Creative Commons licence, unless indicated otherwise in a credit line to the material. If material is not included in the article's Creative Commons licence and your intended use is not permitted by statutory regulation or exceeds the permitted use, you will need to obtain permission directly from the copyright holder. To view a copy of this licence, visit http://creativecommons.org/licenses/by/4.0/.

